# Why are we performing fewer cholecystectomies for mild acute biliary pancreatitis? Trends and predictors of cholecystectomy from the National Readmissions Database (2010–2014)

**DOI:** 10.1093/gastro/goz037

**Published:** 2019-08-29

**Authors:** Sushil Kumar Garg, Fateh Bazerbachi, Shashank Sarvepalli, Shounak Majumder, Shanthi Swaroop Vege

**Affiliations:** 1 Division of Gastroenterology and Hepatology, Mayo Clinic, Rochester, MN, USA; 2 Division of Gastroenterology, Massachusetts General Hospital, Boston, MA, USA; 3 Department of Hospital Medicine, Cleveland Clinic, Cleveland, OH, USA

**Keywords:** cholecystectomy, mild acute biliary pancreatitis, National Readmissions Database, endoscopic retrograde cholangiopancreatography

## Abstract

**Background:**

Current guidelines recommend cholecystectomy for patients with mild acute biliary pancreatitis (MABP) during the index admission because it is associated with better outcomes. In this study, we aimed to assess national trends in cholecystectomy during index admissions for MABP and to identify factors associated with cholecystectomy completion and 30-day readmission.

**Methods:**

Using diagnostic codes and the National Readmissions Database, we identified patients admitted with MABP between 2010 and 2014. Differences in cholecystectomy rates were computed on the basis of various characteristics. We conducted a multivariable analysis to identify factors associated with 30-day readmission and cholecystectomy during the same admission.

**Results:**

We identified 255,695 unique index MABP cases (41.3% male) and the 30-day readmission rate was 12.6%. Overall, 43.8% underwent cholecystectomy and 25% underwent endoscopic retrograde cholangiopancreatography (ERCP) with sphincterotomy. We observed a decreasing trend in both procedures during the study period (*P* < 0.001). In multivariate analysis, odds of 30-day readmission were reduced for patients undergoing ERCP with sphincterotomy (odds ratio, 0.78; 95% confidence interval, 0.74–0.84) or cholecystectomy (odds ratio, 0.37; 95% confidence interval, 0.35–0.39).

**Conclusions:**

For patients with MABP, cholecystectomy or ERCP with sphincterotomy during the index admission decreased the risk of 30-day readmission. Despite this benefit and national guidelines recommending cholecystectomy during the index MABP admission, the rate of cholecystectomies performed nationally decreased during the study period. Further research is needed to understand the implications and reasons underlying this deviation from guidelines.

## Introduction

Gallstones are the most frequent cause of acute pancreatitis (AP) worldwide [1] and up to 8% of those with symptomatic cholelithiasis will have AP. Although most patients with acute biliary pancreatitis (ABP) have mild disease, 20% have severe disease and up to 5% of patients die of ABP [2]. The first-line treatment of ABP is cholecystectomy and the timing of surgery depends on the severity of the ABP. Current guidelines recommend cholecystectomy during the index admission for patients with mild ABP (MABP) [3] and evidence supports the safety of early cholecystectomy in these patients [4].

Multiple studies have reported a lack of adherence to these guidelines [5, 6]. In the USA, the degree of adherence has not been reported in recent years and the impact of cholecystectomy on 30-day readmission rates remains unclear. In this study, we aimed to assess national trends in cholecystectomy completion rates during index admissions for MABP and to identify predictors of 30-day readmission and cholecystectomy in this cohort.

## Materials and methods

### Study design and data source

We conducted a retrospective longitudinal study of admissions to US acute-care hospitals for AP. Data on hospital admissions of all adult patients (age ≥18 years) between 2010 and 2014 were extracted from the National Readmissions Database (NRD). NRD is an inpatient database with several key features—it ‘provides sufficient data for analysis across hospital types and the study of readmissions for relatively uncommon disorders and procedures, [contains] discharge data from 27 geographically dispersed states, accounting for 57.8% of the total US resident population and 56.6% of all US hospitalizations, is designed to support national readmission analyses, and cannot be used for regional, state-, or hospital-specific analyses’. This database tracks patient readmission to same or any other hospital in the USA for every calendar year (1 January through 31 December) but does not link patient data across the preceding or subsequent years. We therefore excluded index admissions that occurred in December from our analysis because readmissions for those cases could not be tracked. The dataset provides de-identified information regarding each admission, including demographics, comorbidities, discharge diagnoses, procedures performed, outcomes, and costs of stay. These databases exclude observation admissions, rehabilitation hospitals, and chemical-dependency units. This study was exempted from IRB review, as no identifiable patient data were included in the database.

### Study population

We used *International Classification of Diseases, Ninth Revision, Clinical Modification* (ICD-9-CM) codes to identify all hospitalized adults (age ≥18 years) with a primary diagnosis of AP (ICD-9-CM code 577.0), a secondary diagnosis of cholelithiasis or choledocholithiasis, and survived to hospital discharge. All diagnostic and procedural codes used for classifications are shown in Supplementary Table 1. AP-related admissions were identified by querying for all diagnostic ICD-9-CM codes corresponding to AP. We excluded patients with acute kidney injury, intensive-care-unit admission, hypotension, shock, and acute hypoxic respiratory failure.

**Table 1.** Baseline characteristics of patients with mild acute biliary pancreatitis after being stratified by readmission and cholecystectomy status

Variable	Overall (*n* = 255,695)	Stratified by readmission			Stratified by cholecystectomy		
		Not readmitted (*n* = 223,369)	Readmitted (*n* = 32,326)	*P*-value	No cholecystectomy (*n* = 143,735)	Cholecystectomy (*n* = 111,960)	*P*-value
Sex, *n* (%)				<0.001			<0.001
Male	105,530 (41.3)	90,831 (40.7)	14,699 (45.5)		64,698 (45.0)	40,832 (36.5)	
Female	150,165 (58.7)	132,538 (59.3)	17,627 (54.5)		79,037 (55.0)	71,128 (63.5)	
Age, *n* (%)				<0.001			
18–44 years	74,750 (29.2)	66,200 (29.6)	8,550 (26.4)		35,889 (25.0)	38,861 (34.7)	
45–64 years	94,358 (36.9)	81,955 (36.7)	12,403 (38.4)		55,115 (38.3)	39,243 (35.0)	
65–84 years	69,713 (27.3)	60,803 (27.2)	8,910 (27.6)		40,319 (28.1)	29,394 (26.3)	
>84 years	16,874 (6.6)	14,411 (6.5)	2,463 (7.6)		12,412 (8.6)	4,462 (4.0)	
Weekend admission, *n* (%)				0.10			<0.001
No	188,097 (73.6)	164,105 (73.5)	23,992 (74.2)		106,739 (74.3)	81,358 (72.7)	
Yes	67,598 (26.4)	59,264 (26.5)	8,334 (25.8)		36,996 (25.7)	30,602 (27.3)	
Hospital size, *n* (%)				<0.001			<0.001
Small	35,747 (14.0)	31,287 (14.0)	4,460 (13.8)		22,157 (15.4)	13,590 (12.1)	
Medium	65,624 (25.7)	58,061 (26.0)	7,563 (23.4)		36,646 (25.5)	28,978 (25.9)	
Large	154,324 (60.4)	134,021 (60.0)	20,303 (62.8)		84,932 (59.1)	69,392 (62.0)	
Hospital type, *n* (%)				0.11			0.14
Government	34,794 (13.6)	30,164 (13.5)	4,630 (14.3)		20,021 (13.9)	14,773 (13.2)	
Private, not-for-profit	182,781 (71.5)	159,895 (71.6)	22,886 (70.8)		102,568 (71.4)	80,213 (71.6)	
Private, profit	38,120 (14.9)	33,310 (14.9)	4,810 (14.9)		21,146 (14.7)	16,974 (15.2)	
Hospital location and teaching status, *n* (%)				<0.001			<0.001
Metropolitan non-teaching	103,015 (40.3)	90,553 (40.5)	12,462 (38.5)		55,417 (38.6)	47,598 (42.5)	
Metropolitan teaching	121,074 (47.3)	105,094 (47.0)	15,980 (49.4)		68,471 (47.6)	52,603 (47.0)	
Nonmetropolitan hospital	31,606 (12.4)	27,722 (12.4)	3,884 (12.0)		19,847 (13.8)	11,759 (10.5)	
Median household income, percentile based on postal code, *n* (%)			0.005			0.005
0–25th percentile	74,842 (29.8)	64,870 (29.5)	9,972 (31.3)		42,737 (30.2)	32,105 (29.1)	
26–50th percentile	64,436 (25.6)	56,526 (25.7)	7,910 (24.8)		36,199 (25.6)	28,237 (25.6)	
51–75th percentile	60,397 (24.0)	52,736 (24.0)	7,661 (24.0)		33,181 (23.5)	27,216 (24.7)	
76–100th percentile	51,863 (20.6)	45,537 (20.7)	6,326 (19.9)		29,209 (20.7)	22,654 (20.6)	
Payer, *n* (%)				<0.001			<0.001
Medicare	95,143 (37.2)	81,334 (36.4)	13,809 (42.7)		59,331 (41.3)	35,812 (32.1)	
Medicaid	36,795 (14.4)	31,243 (14.0)	5,552 (17.2)		20,562 (14.3)	16,233 (14.5)	
Private insurance	86,157 (33.7)	77,312 (34.6)	8,845 (27.3)		43,623 (30.3)	42,534 (38.0)	
Self-pay/other	36,913 (14.4)	32,893 (14.7)	4,020 (12.4)		19,840 (13.8)	17,073 (15.2)	
Disposition, *n* (%)				<0.001			<0.001
Routine	223,401 (87.4)	197,756 (88.5)	25,645 (79.3)		120,821 (84.0)	102,580 (91.6)	
Short-term hospital	2,755 (1.1)	2,146 (1.0)	609 (1.9)		2,479 (1.7)	276 (0.2)	
Skilled nursing facility	11,387 (4.5)	9,357 (4.2)	2,030 (6.3)		7,681 (5.3)	3,706 (3.3)	
Home health care	14,429 (5.6)	11,343 (5.1)	3,086 (9.5)		9,234 (6.4)	5,195 (4.6)	
Against medical advice	3,722 (1.5)	2,766 (1.2)	956 (3.0)		3,518 (2.4)	204 (0.2)	
Comorbidity, *n* (%)							
Myocardial infarction	9,522 (3.7)	7,925 (3.5)	1,597 (4.9)	<0.001	6,333 (4.4)	3,189 (2.8)	<0.001
Congestive heart failure	14,148 (5.5)	11,391 (5.1)	2,757 (8.5)	<0.001	9,615 (6.7)	4,533 (4.0)	<0.001
Peripheral vascular disease	9,317 (3.6)	7,718 (3.5)	1,599 (4.9)	<0.001	6,296 (4.4)	3,021 (2.7)	<0.001
Cerebrovascular disease	4,178 (1.6)	3,513 (1.6)	665 (2.1)	<0.001	2,801 (1.9)	1,377 (1.2)	<0.001
Dementia	2,759 (1.1)	2,310 (1.0)	449 (1.4)	0.002	2,022 (1.4)	737 (0.7)	<0.001
COPD	35,143 (13.7)	29,554 (13.2)	5,589 (17.3)	<0.001	21,857 (15.2)	13,286 (11.9)	<0.001
Rheumatoid disease	4,965 (1.9)	4,045 (1.8)	920 (2.8)	<0.001	3,102 (2.2)	1,863 (1.7)	<0.001
Peptic ulcer disease	4,000 (1.6)	3,295 (1.5)	705 (2.2)	<0.001	2,780 (1.9)	1,220 (1.1)	<0.001
Mild liver disease	30,748 (12.0)	26,366 (11.8)	4,382 (13.6)	<0.001	20,384 (14.2)	10,364 (9.3)	<0.001
Diabetes mellitus	48,833 (19.1)	41,808 (18.7)	7,025 (21.7)	<0.001	30,258 (21.1)	18,575 (16.6)	<0.001
Diabetes mellitus plus complications	5,433 (2.1)	4,317 (1.9)	1,116 (3.5)	<0.001	3,504 (2.4)	1,929 (1.7)	<0.001
Hemiplegia or paraplegia	993 (0.4)	821 (0.4)	172 (0.5)	<0.001	535 (0.4)	458 (0.4)	0.405
Renal disease	16,129 (6.3)	12,831 (5.7)	3,298 (10.2)	<0.001	11,013 (7.7)	5,116 (4.6)	<0.001
Cancer	4,787 (1.9)	3,715 (1.7)	1,072 (3.3)	<0.001	3,414 (2.4)	1,373 (1.2)	<0.001
Moderate or severe liver disease	3,802 (1.5)	2,821 (1.3)	981 (3.0)	<0.001	3,338 (2.3)	464 (0.4)	<.0001
Metastatic cancer	2,083 (0.8)	1,458 (0.7)	625 (1.9)	<0.001	1,725 (1.2)	358 (0.3)	<0.001
AIDS	607 (0.2)	443 (0.2)	164 (0.5)	<0.001	451 (0.3)	156 (0.1)	<0.001
Charlson Comorbidity Index, *n* (%)				<0.001			<0.001
0	129,241 (50.5)	116,428 (52.1)	12,813 (39.6)		63,391 (44.1)	65,850 (58.8)	
1	67,938 (26.6)	59,307 (26.6)	8,631 (26.7)		40,395 (28.1)	27,543 (24.6)	
>1	58,515 (22.9)	47,633 (21.3)	10,882 (33.7)		39,948 (27.8)	18,567 (16.6)	
Secondary diagnoses, *n* (%)							
Cholangitis	6,703 (2.6)	5,765 (2.6)	938 (2.9)	0.06	4,281 (3.0)	2,423 (2.2)	<0.001
Bile-duct obstruction	49,819 (19.5)	40,760 (18.2)	9,059 (28)	<0.001	42,301 (29.4)	7,518 (6.7)	<0.001
Alcohol abuse	29,549 (11.6)	24,342 (10.9)	5,207 (16.1)	<0.001	24,545 (17.1)	5,004 (4.5)	<0.001
Smoking	67,960 (26.6)	58,011 (26)	9,949 (30.7)	<0.001	42,029 (29.2)	25,931 (23.2)	<0.001
Chronic pancreatitis	16,435 (6.4)	12,093 (5.4)	4,342 (13.4)	<0.001	14,674 (10.2)	1,761 (1.6)	<0.001
Procedure or surgery, *n* (%)							
ERCP	63,857 (25.0)	56,112 (25.1)	7,745 (23.9)	0.02	34,759 (24.2)	29,098 (26.0)	<0.001
Percutaneous biliary tube placement	1,367 (0.5)	946 (0.4)	421 (1.3)	<0.001	1,047 (0.7)	320 (0.3)	<0.001

COPD, chronic obstructive pulmonary disease; AIDS, acquired immuno-deficiency syndrome; ERCP, endoscopic retrograde cholangiopancreatography.

### Definitions of variables, comorbidities, and other covariates

The NRD collects demographic information, including age, sex, income, and primary and secondary insurance for all admitted patients. It also contains hospital information (e.g. bed size, location, and teaching status). The Charlson Comorbidity Index (CCI) was calculated and used for comorbidity assessment. To limit our data capture only to patients with MABP (as defined by the revised Atlanta criteria [7]), we restricted our analyses to AP cases that were not associated with organ failure; thus, we evaluated only those cases that did not have acute kidney injury, hypoxia, acute respiratory failure, shock, or death during the index hospitalization. These surrogates were chosen to circumvent the lack of ICD-9 codes for pancreatitis severity in the database. We have provided the definition of hospital size in Supplementary Table 2.

**Table 2.** Most common reasons for readmission of patients with acute mild biliary pancreatitis during the index hospitalization

Reason for readmission	Percentage (*n* = 32,326)
Acute pancreatitis	31.00
Calculus of gallbladder with cholecystitis	3.26
Calculus of gallbladder with acute cholecystitis	3.17
Chronic pancreatitis	2.96
Sepsis	2.55
Cyst and pseudocyst of pancreas	2.54
Calculus of gallbladder without cholecystitis	1.90
Post-operative infection	1.27
Calculus of bile duct without cholecystitis	1.18
Pancreatic cancer	1.09
Pneumonia	0.88

## Outcomes

The primary outcomes of this study were to assess the trend in rates of cholecystectomy and endoscopic retrograde cholangiopancreatography (ERCP) with sphincterotomy in patients admitted with MABP to US hospitals. We performed a multivariate regression analysis to (i) identify predictors of index cholecystectomy in patients with MABP and (ii) identify predictors of all-cause 30-day readmission in these patients.

## Statistical analysis

The statistical analysis described below was published previously [8] and is reused here under Creative Commons license. Data are presented as mean ± standard deviation (SD) for continuous variables or weighted frequency (%) for categorical variables. A univariate analysis was performed to assess differences between the two groups (no cholecystectomy during index admission vs performance of the procedure during index admission); continuous variables were compared by using *t*-tests and categorical variables were compared by using χ^2^ tests. In addition, multivariable analysis was used to assess differences between the groups in terms of the outcomes of interest while adjusting for patient and hospital characteristics. Survey logistic-regression analysis was used to model 30-day readmission risk and cholecystectomy. We used the Cochran-Armitage test to assess trends in cholecystectomy. The NRD is based on a complex sampling design that includes stratification, clustering, and weighting; SAS Survey procedures facilitate the unbiased assessment of population estimates. *P*-values <0.001 were considered statistically significant because of the large sample size; this significance criterion has been used by previous National Inpatient Sample studies. All analyses were performed with SAS (version 9.4; SAS Institute Inc., Cary, NC).

## Results

### Baseline patient characteristics

**Figure 1.**
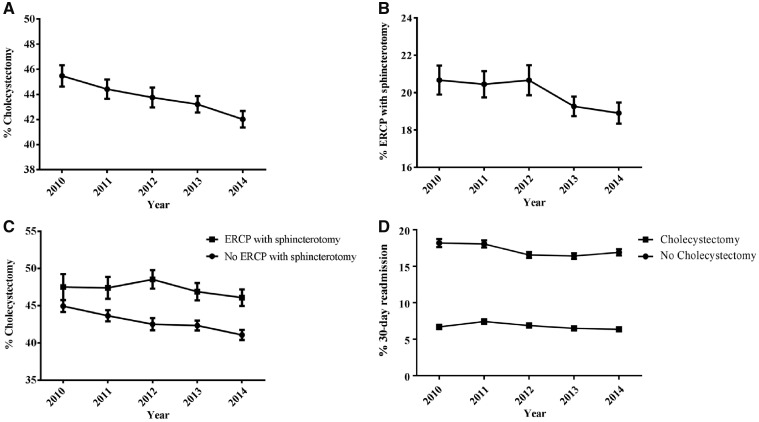
Outcomes of patients with mild acute biliary pancreatitis (MABP), 2010–2014. (A) Trends of cholecystectomy. (B) Trends of endoscopic retrograde cholangiopancreatography (ERCP) with sphincterotomy in MABP. (C) Rates of cholecystectomy, stratified by ERCP with sphincterotomy status. (D) 30-day readmission rate, stratified by cholecystectomy status.

We identified 255,695 index MABP discharges during the study period. Baseline characteristics of these patients, stratified by readmission and cholecystectomy status, are listed in Table 1. Of the 255,695 patients identified with an index admission of MABP, 32,326 (12.6%) were readmitted within 30 days. Figure 1A and B shows decreasing trends in cholecystectomy and ERCP with sphincterotomy during this period (*P* < 0.001). Overall, 45.4% of patients underwent cholecystectomy and 20.6% underwent ERCP with sphincterotomy in 2010; in 2014, 42.0% and 18.6% underwent cholecystectomy and ERCP with sphincterotomy, respectively. Figure 1C shows differences in cholecystectomy rates for patients who did vs did not undergo ERCP with sphincterotomy. Figure 1D shows differences in 30-day readmission rates for patients who did vs did not undergo cholecystectomy during the index admission. Table 2 lists the most common reasons for readmission in patients with index admissions of MABP.

### Factors associated with readmission and cholecystectomy

We performed multivariate logistic-regression analysis to identify predictors of 30-day all-cause readmission and factors associated with cholecystectomy during the index admission for patients admitted with MABP (Table 3):

- The odds of all-cause readmission and cholecystectomy decreased with patient age. Patients admitted to a large hospital [odds ratio (OR), 1.11; 95% confidence interval (CI), 1.03–1.19] were more likely to be readmitted within 30 days. Those admitted to medium (OR, 1.25; 95% CI, 1.15–1.36) or large hospitals (OR, 1.31; 95% CI, 1.21–1.41) were more likely to undergo cholecystectomy than those admitted to a small hospital.
- Compared with patients who had a routine discharge home, patients who were discharged to a short-term hospital (OR, 1.68; 95% CI, 1.4–2.01), to a skilled nursing facility (OR, 1.14; 95% CI, 1.02–1.28), with home health care (OR, 1.51; 95% CI, 1.38–1.65), or against medical advice (OR, 1.79; 95% CI, 1.55–2.07) had significantly higher odds of readmission. Patients who underwent ERCP with sphincterotomy or cholecystectomy on index admission had decreased odds of readmission (OR, 0.78; 95% CI, 0.74–0.84 and OR, 0.37; 95% CI, 0.35–0.39, respectively). Patients with higher CCI or alcohol abuse were less likely to undergo cholecystectomy.

**Table 3.** Predictors of 30-day readmission and cholecystectomy for patients admitted with mild acute biliary pancreatitis

Variable	Comparator vs reference	30-day readmission		Cholecystectomy	
		Odds ratio (95% CI)	*P*-value	Odds ratio (95% CI)	*P*-value
Sex	Female vs male	0.938 (0.896–0.982)	0.006	1.156 (1.116–1.197)	<0.001
Age group, years	45–64 vs 18–44	0.930 (0.876–0.987)	0.02	0.771 (0.741–0.802)	<.001
	65–84 vs 18–44	0.698 (0.640–0.761)	<0.001	0.881 (0.831–0.934)	<0.001
	>84 vs 18–44	0.642 (0.572–0.719)	<0.001	0.410 (0.377–0.446)	<0.001
Payer	Medicaid vs Medicare	0.967 (0.886–1.054)	0.44	1.144 (1.076–1.217)	<0.001
	Private insurance vs Medicare	0.722 (0.668–0.781)	<0.001	1.356 (1.285–1.431)	<0.001
	Self-pay/another vs Medicare	0.699 (0.635–0.769)	<0.001	1.333 (1.248–1.424)	<0.001
Median household income as percentile based on postal code	26th–50th vs 0–25th percentile	0.951 (0.889–1.017)	0.14	0.994 (0.946–1.045)	0.83
	51th–75th vs 0–25th percentile	1.037 (0.974–1.103)	0.26	0.997 (0.946–1.052)	0.92
	76th–100th vs 0–25th percentile	1.003 (0.935–1.076)	0.94	0.916 (0.860–0.976)	0.007
Weekend admission	Yes vs no	0.994 (0.947–1.043)	0.81	1.104 (1.069–1.142)	<0.001
Hospital size	Medium vs small	0.950 (0.876–1.031)	0.22	1.250 (1.149–1.359)	<0.001
	Large vs small	1.106 (1.029–1.189)	0.006	1.305 (1.210–1.408)	<0.001
Hospital type	Private, not-for-profit vs government	0.944 (0.883–1.010)	0.10	1.066 (0.988–1.150)	0.10
	Private, for-profit vs government	0.992 (0.911–1.081)	0.85	1.077 (0.986–1.177)	0.10
Teaching status of hospital	Metropolitan teaching vs metropolitan non-teaching	0.944 (0.896–0.994)	0.03	1.137 (1.070–1.208)	<0.001
	Nonmetropolitan hospital vs metropolitan non-teaching	0.884 (0.811–0.963)	0.005	0.788 (0.722–0.861)	<0.001
Length of stay, days	>7 vs <7	1.791 (1.703–1.883)	<0.001	1.578 (1.514–1.645)	<0.001
Disposition	Short-term hospital vs routine	1.680 (1.404–2.011)	<0.001	–	–
	Skilled nursing facility vs routine	1.140 (1.020–1.275)	0.02	–	–
	Home health care vs routine	1.511 (1.383–1.652)	<0.001	–	–
	Against medical advice vs routine	1.789 (1.548–2.069)	<0.001	–	–
Charlson Comorbidity Index	1 vs 0	1.150 (1.086–1.218)	<0.001	0.753 (0.727–0.780)	<0.001
	>1 vs 0	1.575 (1.482–1.674)	<0.001	0.532 (0.509–0.555)	<0.001
Alcohol abuse	Yes vs no	1.150 (1.086–1.218)	<0.001	0.222 (0.210–0.236)	<0.001
ERCP with sphincterotomy	Yes vs no	0.783 (0.735–0.835)	<0.001	1.007 (0.962–1.054)	0.76
Cholecystectomy	Yes vs no	0.371 (0.352–0.391)	<0.001	–	–

CI, confidence interval; ERCP, endoscopic retrograde cholangiopancreatography.

## Discussion

Cholecystectomy is recommended following an MABP episode to prevent the development of biliary-related complications based on a PONCHO trial that was very well done [9]. The PONCHO trial was a multicenter study performed in 23 hospitals in the Netherlands that randomly assigned 266 inpatients to interval cholecystectomy or same-admission cholecystectomy. The primary endpoint of this study was recurrent gallstone-related complications within 6 months. This study found that that interval group had 17% gallstone-related complications compared to 5% in the same-admission group. We also observed that those who underwent cholecystectomy during the index admission were less likely to be readmitted than patients who did not. This difference is a cause for concern from a health-care-use standpoint. Although our findings are original in terms of their pertaining to the US health-care system, analogous observations have been made in other countries [10].

Our study used multivariate regression analyses to show that multiple factors affected the odds of cholecystectomy during the index admission. In terms of demographic factors, cholecystectomies were mainly undertaken in younger patients, especially those aged 18–44 years. This higher rate may be attributable to the younger group’s perceived better medical fitness for surgery. Women were more likely to undergo cholecystectomy during the same admission for MABP and were subsequently less likely to be readmitted for any cause. The higher rate of surgery for women may be associated with surgeon preference, with previous research showing less complicated surgery in women [11]. In terms of hospitalization factors, weekend admissions were associated with higher rates of cholecystectomy and subsequently lower rates of readmission. Previous research has shown similar rates of surgical complications after weekend and weekday cholecystectomies [12]. Although there has been a slight decrease in the rate of cholecystectomy and ERCP over the course of this study, we did not see an increase in readmission for unclear reason.

Our study confirms that index cholecystectomy is the single most effective intervention for decreasing the odds of all-cause and MABP-specific readmissions. We have previously shown that, when patients are stratified by the cause of pancreatitis, only those with gallstone pancreatitis accrued a statistically significant decrease in readmission rates after the index admission [13]. We have also observed that undergoing ERCP with sphincterotomy is associated with a modest decrease in the readmission rate. These findings are congruent with previous research that showed a mitigating but not terminating effect of sphincterotomy on the recurrence of gallstone pancreatitis [14].

The reasons for delayed cholecystectomy, even when it is clearly indicated, are multifaceted. First, there is a pervasive belief among physicians regarding the danger of the procedure shortly after recovery from an AP episode. This notion has been challenged by research reporting no difference in technical complexity between early and delayed cholecystectomy, even when cholecystitis is the indication for the operation, which theoretically has more surgical complications in the setting of an acutely inflamed gallbladder [15]. Second, economic restraints and a changing reimbursement landscape may prompt emphasis on shorter hospitalizations. Third, although guidelines and recommendations may appear ideal from a patient-outcome perspective, their implementation may not be practical or realistic in all settings such as safety-net hospitals or other facilities with limited resources [16].

One potential solution is the implementation of a medical discharge−surgical readmission pathway, which would plan for elective outpatient cholecystectomy within 2 weeks after discharge from an MABP admission. In this paradigm, medical and anesthesia pre-operative assessment, as well as informed consent for the surgery, are finalized before discharge [17]. Another option entails direct admission to a surgical service, which was previously reported to increase the rate of index cholecystectomy and decrease the risk of readmission [18].

Although our study offers substantial strength from the large sample size and allows identification of robust associations, we nonetheless acknowledge numerous inherent and unavoidable limitations. First, we conducted a retrospective, uncontrolled study that did not allow inference of causality and at best was an exploratory examination of associations. Second, ICD-9 coding almost certainly includes inaccuracies, which are common with any administrative database. Third, the scope of our examination is limited by critical absences in clinical data, such as anthropomorphic characteristics (e.g. body mass index) and laboratory values (e.g. C-reactive protein). Lastly, the limited period of data collection (before publication of the pivotal PONCHO study in 2015) may not portray changes in practice that hopefully are influenced by the dissemination of findings from that well-designed trial. Another limitation of our study is that some of the readmissions could have been related to bile-ductal leaks or pancreatic necrosis, which we are unable to tell with confidence because of the known difficulty in looking at large administrative databases retrospectively.

Although the trends observed in our current analysis suggest considerable deviation from accepted guidelines, the practice may change according to that high-level evidence and the publication of new guidelines.

## Conclusions

For patients with MABP, cholecystectomy during the index admission was the strongest predictive factor associated with a decreased risk of readmission. However, the proportion of patients undergoing these procedures steadily decreased from 2010 to 2014. This decrease could not be explained by increasing disease severity or comorbidity. Our research emphasizes the need for policies to encourage providers to perform cholecystectomy during the index admission, as highlighted by high-quality evidence in the literature.

## Authors’ contributions

S.K.G.: study concept, study design, analysis, writing of the manuscript; F.B.: writing of the manuscript; S.S.: writing of the manuscript, analysis; S.M.: study design, critical edits; S.S.V.: study design, critical edits.

## Conflicts of interest

The authors declare that there are no potential conflicts of interest.

## Supplementary Material

goz037_Supplementary_Tables
