# Supplementary material for: Why are we performing fewer cholecystectomies for mild acute biliary pancreatitis? Trends and predictors of cholecystectomy from the National Readmissions Database (2010–2014)
Source: Gastroenterol Rep (Oxf). 2019 Aug 29;7(5):331–7. doi: 10.1093/gastro/goz037 (PMC6821273; doi:10.1093/gastro/goz037)
Supplement: goz037_Supplementary_Tables [file goz037_supplementary_tables.docx]

**Table 1.** ICD-9-CM codes for relevant conditions and procedures.

| **Condition or Procedure** | **Code** |
| --- | --- |
| Acute pancreatitis | 577.0 |
| Cholelithiasis | 574, 574.00, 574.01, 574.10, 574.11, 574.20, 574.21, 574.30, 574.40, 574.41, 574.50, 574.51, 574.60, 574.61, 574.70, 574.71, 574.80, 574.81, 574.90, 574.91 |
| Choledocholithiasis | 576.2, 576.8, 782.4, 576.9 |
| Cholecystectomy | 51.21, 51.22, 51.23, 51.24 |
| ERCP with sphincterotomy | 51.85 |
| Percutaneous biliary drainage | 51.01, 51.96, 51.98 |
| Chronic pancreatitis | 577.1 |
| Cholangitis | 576.1 |
| Alcohol abuse | 29.0, 291.1, 291.2, 291.3, 291.4, 291.5, 291.81, 291.82, 291.89, 291.9, 303.00, 303.02, 303.03, 303.90, 303.91, 303.92, 303.93, 305.00, 305.02, 305.03, 760.71, 980.0, 357.5, 425.5, 535.30, 535.31, 571.0, 571.1, 571.2, 571.3 |
| Smoking | 305.1, V158.2 |
| Acute respiratory failure | 518.0, 518.81. 518.82, 518.84 |
| Acute kidney injury | 584.5, 584.6, 584.7, 584.8, 584.9 |
| Morbid obesity | 278.01, V854, V854.1, V854.2, V854.3, V854.4 |

ICD-9-CM, *International Classification of Diseases, Ninth Revision, Clinical Modification*; ERCP, endoscopic retrograde cholangiopancreatography;

**Table 2**. Definition of hospital size

| **BEDSIZE CATEGORIES (Beginning in 1998)** | | | |
| --- | --- | --- | --- |
| **Location and Teaching Status** | **Hospital Bedsize** | | |
|  | **Small** | **Medium** | **Large** |
| **NORTHEAST REGION** | | | |
| Rural | 1-49 | 50-99 | 100+ |
| Urban, nonteaching | 1-124 | 125-199 | 200+ |
| Urban, teaching | 1-249 | 250-424 | 425+ |
| **MIDWEST REGION** | | | |
| Rural | 1-29 | 30-49 | 50+ |
| Urban, nonteaching | 1-74 | 75-174 | 175+ |
| Urban, teaching | 1-249 | 250-374 | 375+ |
| **SOUTHERN REGION** | | | |
| Rural | 1-39 | 40-74 | 75+ |
| Urban, nonteaching | 1-99 | 100-199 | 200+ |
| Urban, teaching | 1-249 | 250-449 | 450+ |
| **WESTERN REGION** | | | |
| Rural | 1-24 | 25-44 | 45+ |
| Urban, nonteaching | 1-99 | 100-174 | 175+ |
| Urban, teaching | 1-199 | 200-324 | 325+ |
